# Primitive and Secondary Sutures of the Soft Parts

**Published:** 1918-12

**Authors:** 


**Primitive and Secondary Sutures of the Soft Parts in 115 Wounds with Fractures.** By MM. THEVENOT and TH. TUFFIER. *Bulletin de la Société de Chirurgie de Paris,* June 18, 1918.

The suture of the soft part, when the injury includes some fracture, is not considered as an advisable intervention by most surgeons. Nevertheless the authors have performed it in 115 cases of severe fractures of the bone shafts.

The remote results of these interventions are summarized as follows:

88 sutures were primitive ones and were performed on arm, fore-arm, thigh and leg. Among these, 50 results were excellent; 30 were considered as disjunctions, even if no functional disturbances appeared subsequently; 8 results were unknown.

In 27 cases secondary sutures were performed, giving 17 excellent results, 4 fair ones and 4 unknown.

The authors consider that sutures should be resorted to in cases of fractures as well as in cases of wounds strictly limited to the soft parts.

The operative mode adopted by the authors was the following one. The patient was radiographed, and anesthetics were administered immediately. The bruised muscles and aponeurosis were excised; but this excision was carefully limited to the injured parts, and stopped as soon as sound tissues were found. As for the bone, the splinters were removed and the marrow cleansed. After a swabbing with ether, the wound was sutured.

If the wound appeared too infected to admit of immediate suture, it was left widely open, and treated with Dakin's solution, or injected twice a day with ether, after fixing up of Carrel's tubes.

When bacteriological control found no more streptococci, secondary suture was performed. In some cases the loss of skin was such that grafting proved necessary.

The authors consider that all wounds with fractures must be closed as soon as the injury proves aseptic. The wounds by bullets or shell-fragments presenting punctiforn orifices act spontaneously as closed wounds. But in all other cases sutures are advisable.

Should this suture be a primitive or a secondary one, no general answer can be given. It depends entirely on the kind of wound, its extent, and the number and kind of the projectiles; it also depends very much on the injury of the bone, the number of splinters and the possibilities of secondary infection.

But after the wound is sutured it appears to improve quickly, and therefore this method of treatment should spread widely.

